# Retraction: Mutant p53 determines pancreatic cancer poor prognosis to pancreatectomy through upregulation of cavin-1 in patients with preoperative serum CA19-9 ≥ 1,000 U/mL

**DOI:** 10.1038/srep25115

**Published:** 2016-05-13

**Authors:** Jin-Feng Xiang, Wen-Quan Wang, Liang Liu, Hua-Xiang Xu, Chun-Tao Wu, Jing-Xuan Yang, Zi-Hao Qi, Ya-Qi Wang, Jin Xu, Chen Liu, Jiang Long, Quan-Xing Ni, Min Li, Xian-Jun Yu

Scientific Reports 6: Article number: 19222; 10.1038/srep19222 published online: 01122016; updated: 05132016.

The authors of this Article have requested that it is retracted. In the western blotting analysis, the rabbit monoclonal antibody to human mutant p53 rather than wild type p53 (1:1000, ab32049, Abcam) was used. While in the immunohistochemistry detection, the mouse monoclonal antibody to both wild-type and mutant forms of human p53 (1:100, ab28, Abcam) was used. The authors believe that the use of antibodies for both wild type p53 and mutant p53 is not appropriate because it cannot reflect the overexpression status of mutant TP53. The results as described in the manuscript stated that the number of patients with overexpression of mutant p53 was 84/241 (34.9%), using the mouse monoclonal antibody to both wild-type and mutant forms of human p53 (1:100, ab28, Abcam). Ninety (37.3%) pancreatic ductal adenocarcinomas (PDACs) showed a virtual absence of p53 immunolabeling compared with the adjacent normal tissues. These patients were also considered to be “abnormal” in p53 immunolabeling. In contrast, the normal immunolabeling of p53 was detected in 67 (27.8%) of the 241 PDACs. However, the authors later found that the number of patients with overexpression of mutant p53 was actually 113/241 (46.9%) in these same specimens when using the specific anti-mutant p53 antibody (1:100, ab32049, Abcam). This inconsistency could impact the subsequent analyses and the previous conclusion of this study could not be drawn. Therefore, the authors feel they cannot guarantee the accuracy of the results and conclusions described in this paper, and believe it is necessary to retract it.

